# IL6 Induces mtDNA Leakage to Affect the Immune Escape of Endometrial Carcinoma via cGAS-STING

**DOI:** 10.1155/2022/3815853

**Published:** 2022-06-02

**Authors:** Xue Zeng, Xiaosong Li, Yundong Zhang, Chaoxia Cao, Qin Zhou

**Affiliations:** ^1^Department of Neurology, The Third Affiliated Hospital of Chongqing Medical University, Chongqing 401120, China; ^2^Department of Gynaecology, Qianjiang Central Hospital of Chongqing, Chongqing 409090, China; ^3^Department of Obstetrics and Gynecology, The First Affiliated Hospital of Chongqing Medical University, Chongqing 400016, China

## Abstract

Endometrial carcinoma (EC) is a commonly diagnosed gynecological malignancy. Interleukin-6 (IL6) plays a critical role in modulating the progression of several types of tumors, including EC. However, the specific mechanism of IL6 in regulating EC progression has not been clearly elucidated. In this study, we performed a series of functional experiments to explore the potential mechanisms involved in IL6 function in the progression of EC. Here, we found that IL6 increased reactive oxygen species (ROS) generation by enhancing the NADPH oxidase (NOX) level and induced mtDNA leakage in EC cells, which further caused the activation of the downstream cGAS-STING signaling and increased production of extracellular vesicle (EV) production from EC cells. Besides, the activation of cGAS-STING signaling enhanced the expression of type I IFN and its downstream molecule PD-L1 through the TBK1-IRF3 pathway. Importantly, a high level mtDNA and PD-L1 were present in EVs derived from IL6-induced EC cells; these vesicles were shown to be able to induce T cell apoptosis. Finally, anti-PD-L1 treatment in mice showed that blockade of PD-L1 significantly reversed tumor immune escape mediated by IL6-induced EVs. Together, we provide evidence that IL6 induced mtDNA leakage to regulate the immune escape of EC cells. Our findings may provide a novel clue for the development of therapeutic targets for EC.

## 1. Introduction

Endometrial carcinoma (EC) is a worldwide gynecological malignancy with increasing morbidity and mortality [1]. EC is normally diagnosed early and has a favorable prognosis, while a fraction of patients develop metastatic or recurrent diseases that are not suitable for local treatment [2]. There are many causes of EC. Recent studies have shown that oxidative stress was a crucial factor in the progression of EC [3]. Besides, the PD-1/PD-L1 checkpoint has also been demonstrated to play a vital role in recurrent or metastatic EC [4].

Cytokines are small proteins (15~20 kD) with diverse functions in the regulation of immunity, development, metabolism, aging, and cancer [5]. As a multifunctional factor, IL6 family showed multipotency in different life processes [6]. The dysregulation of IL6 expression has been reported to promote cancer progression in different ways, mainly through direct and intrinsic effects on cancer cell viability and indirect effects of the local tumor microenvironment on stromal cells by regulating inflammation, immunosuppression, and angiogenesis [7, 8]. Furthermore, abnormal expression of IL6 has been confirmed to be associated with reactive oxygen species (ROS) production [9, 10]. The deterioration of many cancers is accompanied by an increase in ROS. An excess of ROS has been identified to activate protumor signals, enhance cell survival and proliferation, and drive DNA damage and genetic instability [11]. Besides, ROS level contribute to the destruction of mitochondria, thus promoting the accumulation of potentially destructive levels including ROS and Ca^2+^ [12].

Mitochondria are unique organelles because they have their own genomes, which are different from the nuclear genomes. Compared with nuclear DNA, mitochondrial DNA is more fragile [13]. Radiation, toxic chemicals, the infection of pathogenic microorganisms, gene mutation, and ROS level are all factors leading to mitochondrial stress. Stimulated with stress, mitochondria release mtDNA to self-repair, triggering the innate immune response [14]. The cGAS-STING signaling axis has been reported to be an essential DNA sensing mechanism in innate immunity and virus defense. Current studies have shown that cGAS-STING signaling is activated in highly aggressive tumors and is closely involved in tumor progression. Meanwhile, increasing evidence indicate that the cGAS-STING pathway is involved in tumor promotion and metastasis and its chronic activation can induce immunosuppressive tumor microenvironment [15, 16]. STING agonist treatment has been shown to significantly increase PD-L1 expression and the release of proinflammatory cytokines in cancer cells [17].

Programmed death 1 (PD-1) and its ligand PD-L1 play a vital role in maintaining physiological immune homeostasis. Nevertheless, the tumor will usurp PD-1/PD-L1 axis to weaken antitumor immunity and promote chronic infection and tumor survival [18]. Furthermore, exo-PD-L1 is produced by mounting tumors for immune escape, thus enhancing their ability to proliferate [19].

Herein, we found that IL6 induced EC cells to produce ROS. Dysregulation of ROS led to leakage of mtDNA and triggered the downstream cGAS-STING signaling, thus increasing the level of PD-L1. Further, the expression levels of mtDNA and PD-L1 were significantly higher in EVs derived from IL6-induced EC cells. And these EVs were found to induce T cell apoptosis and tumor immune escape. Taken together, our study revealed a novel mechanism by which IL6 induced mitochondrial mtDNA leakage, which in turn affected tumor immune escape. And it might provide a possibility of using EVs containing mtDNA and PD-L1 as biomarkers for EC diagnosis.

## 2. Materials and Methods

### 2.1. Animals

Mice (C57BL/6, females) were purchased from Shanghai Animal Laboratory Center and housed in the Third Affiliated Hospital of Chongqing Medical University, Chongqing. All animal experiments complied with ethical regulations and were approved by the Medical Ethics Committee of the Third Affiliated Hospital of Chongqing Medical University, Chongqing. In the first experiments, 6 mice were first subcutaneously injected with 4 × 10^6^ MFE-296 cells. After 2 weeks of injection, the mice were equally divided into two groups (*n* = 3 in each group) and intravenously treated with the same dose of IL6 (2.5 *μ*g/kg) or an equal volume of PBS every 7 days for 28 days. 7 days after the last injection treatment, all mice were euthanized. In the second experiments, 6 mice were firstly subcutaneously injected with MFE-296 cells. After 7 days of injection, the mice were equally divided into two groups (*n* = 3 in each group), and the mice subcutaneously injected with same dose of EVs/IL6 or anti-PD-L1-masking EVs/IL6 every 7 days for 28 days. 7 days after the last injection treatment, all mice were euthanized. Tumor lengths and widths were measured using calipers. Tumor volumes were calculated using *V* (mm^3^) = (*L* × *W*^2^)/2, where *L* is the tumor length and *W* is the tumor width). This study was performed in strict accordance with the requirements in the Guide for the Care and Use of Laboratory Animals of the National Institutes of Health.

### 2.2. Cell Culture

EC cell lines (MFE-296) were purchased from ECACC (Public Health England Culture Collections, UK). All cells were cultured in the RPMI-1640 medium with 10% FBS and 1% penicillin-streptomycin. The cell lines were cultured in an incubator at 37°C and 5% CO_2_.

### 2.3. Real-Time Reverse Transcription-PCR (qRT-PCR)

The cells were planted in 24-well plates and treated according to the experimental design. Cells were washed with PBS and total RNA was isolated using TRIzol reagent (Life Technologies, NY, USA) according to the manufacturer's instructions. SuperScript II (Invitrogen-Life Technologies, Carlsbad, CA, USA) was used for reverse transcription. qRT-PCR was performed using the SYBR PrimeScript RT-PCR kit (Takara, Japan) according to the manufacturer's instructions. For analysis, mRNA expression levels were standardized against glyceraldehyde-3-phosphate dehydrogenase (GAPDH) mRNA measured for each sample. The relative expression of all genes was calculated and normalized using the 2^−*ΔΔ*Ct^ relative to GAPDH. Specific primers are shown: GAPDH: 5′-ACCCTTTGGACGCACGGCAT-3′, 5′-GCCAGCCTCTCCTGATTTTAGTGT-3′; Hk2: 5′- GGGAACACAAAAGACCTCTTCTGG-3′, 5′-CCGGCTGCGTATTCTACGTT-3′; Ptger2: 5′-CCTGCTGCTTATCGTGGCTG-3′, 5′-GCCAGGAGAATGAGGTGGTC-3′; Nduf1: 5′-CTTCCCCACTGGCCTCAAG-3′, 5′-CCAAAACCCAGTGATCCAGC-3′; Tert1: 5′-CTAGCTCATGTGTCAAGACCCTCTT-3′, 5′-GCCAGCACGTTTCTCTCGTT-3′; ND1: 5′-CTAGCAGAAACAAACCGGGC-3′, 5′-CCGGCTGCGTATTCTACGTT-3′; ND4: 5′-AACGGATCCACAGCCGTA-3′, 5′-AGTCCTCGGGCCATGATT-3′; Cybt1: 5′-GCTTTCCACTTCATCTTACCATTTA-3′, 5′-TGTTGGGTTGTTTGATCCTG-3′; DLOOP: 5′-TTTAGACGGGCTCACATCACC-3′, 5′-TGCGTGCTTGATGCTTGTC-3′; IFN-*α*: 5′-GGATGTGACCTTCCTCAGACTC-3′, 5′-ACCTTCTCCTGCGGGAATCCAA-3′; IFN-*β*: 5′-ATGACCAACAAGTGTCTCCTCC -3′, 5′-GGAATCCAAGCAAGTTGTAGCTC-3′; IRF3: 5′-TACCAAGGCCCTGAGGCAC-3′, 5′-GGAATCCAAGCAAGTTGTAGCTC-3′; and PD-L1: 5′-GACCAGCTTTTGAAGGGAAATG-3′, 5′-CTGGTTGATTTTGCGGTATGG-3′.

### 2.4. NADH Oxidase (NOX) Activity Assay

We performed NOX Activity Assay kit to detected NOX activity according to the manufacturer's protocol (Sangon, China). Briefly, NOX can oxidize NADH to NAD, and the oxidation of NADH is coupled with the reduction of 2,6-dichlorophenol indigo blue (DCPIP), and the blue DCPIP is reduced to colorless DCPIP. The reduction rate of blue DCPIP was measured at 600 nm, and the activity of NADH oxidase was calculated.

### 2.5. ROS Detection Assay

We performed the Cellular ROS Assay kit to detected cellular ROS content according to the manufacturer's protocol (Abcam ab113851, Cambridge, MA, USA). Briefly, cells in suspension in tubes/seeds were collected and adherent cells are allowed to attach to a 96-well plate. We stained with DCFDA for 30 min (suspension)/45 min (adherent) after washing in buffer. If the cells were in suspension after washing in buffer, they were transferred to a microplate and used them for analysis with a microplate reader.

### 2.6. Western Blot

Total proteins were extracted using cell lysis buffer (BioFeng, Changsha, Hunan, China). After the protein concentrations were determined by BCA kits, the protein samples were extracted and separated by 10% SDS-PAGE gel and then transferred to a PVDF membrane (Millipore, USA). The membrane was then blocked with 5% skimmed milk and incubated overnight, using the following main detection antibodies at 4°C: anti-GAPDH (1 : 1,000; Abcam), anti-Histon-H3 (1 : 1,000; Abcam), anti-Hsp60 (1 : 1,000; Abcam), anti-cGAS (1 : 1,000; Abcam), anti-STING (1 : 1,000; Abcam), anti-pSTING (1 : 1,000; Abcam), anti-TBK1 (1 : 1,000; Abcam), anti-pTBK1 (1 : 1,000; Abcam), anti-IRF3 (1 : 1,000; Abcam), anti-pIRF3 (1 : 1,000; Abcam), anti-PD-L1 (1 : 1,000; Abcam), anti-CD9 (1 : 1,000; Abcam), anti-TSG101 (1 : 1,000; Abcam), anti-Alix (1 : 1,000; Abcam), anti-Hsp90 (1 : 1,000; Abcam), or anti-*β*-actin (1 : 5,000; Proteintech). We washed 3 times with TBS-T, and the membranes were cultured with the secondary antibody at 24°C for 1 hr. The western blots were pictured using an ECL Reagent (Pierce, USA), and the density was verified using ImageJ software (NIH, USA).

### 2.7. ELISA

After ventilation, right lung tissue was collected and ground, followed by centrifugation to collect supernatant. The prepared well plate was added with 100 *μ*l per well, incubated at 37°C for 90 min. The biotin anti-human PD-L1 antibody working solution of 100 *μ*l was added to each well and reacted at 37°C for 60 min. Concentrations of PD-L1 (Abcam, ab214565) were detected by ELISA kits. All operations are strictly in accordance with kit instruction.

### 2.8. EV Purification

We performed ultracentrifugation to purify EC-derived EVs. EVs were cultured in Ultra medium (Lonza, USA, 12-725 F) containing 10% EVs-free FBS (System Biosciences, USA, 50A-1) for 72 hr prior to isolation. Briefly, the supernatants were centrifuged at 300 g for 10 min, 2000 g for 10 min, and 10,000 g for 30 min at 4°C to eliminate cell debris. The supernatant was precipitated by centrifugation at 110,000 g for 70 min. The EV precipitate was washed in 3 ml PBS, then recentrifuged at 110,000 g for 70 min, and resuspended in PBS.

### 2.9. Transmission Electron Microscopy (TEM)

We used differential centrifugation to obtain EVs secreted by EC. The separated EVs were diluted with PBS and then fixed in 2% paraformaldehyde. Then, the samples were transferred to a formvar copper mesh. It was blotted and contrast stained with uranyl acetate (Electron Microscopy Sciences, USA) at room temperature and then blotted on filter paper and air-dried before analysis. The FEI Tecnai 110 kV transmission electron microscope was used to examine the formulation at 80 kV.

### 2.10. Nanoparticle Tracking Analysis (NTA)

We used differential centrifugation to obtain EC-derived EVs. EVs were purified by ultracentrifugation and quantified using the NanoSight NS300 (NanoSight, UK). The sample was diluted to the optimal concentration in PBS. Each sample was recorded 3 times for 30 seconds, the temperature was manually monitored, and the camera level was set to 10. Data were represented as mean ± SD of three replicates. All measurements were performed at room temperature.

### 2.11. Immunofluorescence Staining of ROS

After treated with IL6 or H_2_O_2_, MFE-296 cells were fixed in 4% paraformaldehyde dissolved in PBS. Cells were permeabilized using 0.1% Triton X-100 on ice for 30 minutes; then, cells were blocked with 4% bovine serum for 1 hour at room temperature. Then, cells were incubated with 5 *μ*mol/l MitoSOX (Thermo Fisher Scientific, USA), for 10 minutes in a dark humidified chamber. Cell nuclei were stained with 4′,6-diamidino-2-phenylindole (DAPI) at room temperature. Immunostaining images were collected using a confocal microscopy.

### 2.12. Immunofluorescence Staining of Cytosolic dsDNA

After treatment with IL6 or IL6+NAC, MFE-296 cells were fixed in 4% paraformaldehyde dissolved in PBS. Cells were permeabilized using 0.1% Triton X-100 on ice for 30 minutes; then, cells were blocked with 4% bovine serum for 1 hour at room temperature. Then, cells were incubated with primary anti-dsDNA (1 : 1000, Sigma) and anti-Hsp60 (1 : 1000, Proteintech), for 3 hours at room temperature, followed by incubation with FITC-labeled anti-IgG (1 : 500, Sigma) and Cy3-labeled secondary antibodies for an hour. Immunostaining images were collected using a confocal microscopy. Antibodies used to stain dsDNA have been validated in a previous study [20].

### 2.13. Immunofluorescence Staining of T Cells

After treated with EVs/NC, EVs/IL6, or EVs/IL6+Ethbr, MFE-296 cells were fixed in 4% paraformaldehyde dissolved in PBS. Cells were permeabilized using 0.1% Triton X-100 on ice for 30 minutes; then, cells were blocked with 4% bovine serum for 1 hour at room temperature. Then, cells were incubated with primary anti-mouse CD3 (1 : 1000, Sigma) and anti-CD8 (1 : 1000, Sigma), for 3 hours at room temperature, followed by incubation with FITC-labeled anti-IgG (1 : 500, Sigma) and Cy3-labeled secondary antibodies for an hour. Cell nuclei were stained with 4′,6-diamidino-2-phenylindole (DAPI) at room temperature. Immunostaining images were collected using a confocal microscopy.

### 2.14. Flow Cytometry

We extracted T cells from mouse splenocytes using a Mouse T Lymphocyte Enrichment Set-DM according to the manufacturer's instructions. After that, cells were incubated with anti-mouse CD3, anti-mouse CD4, anti-mouse CD8, and anti-mouse CD25 (STEMCELL Technologies Inc., Vancouver, Canada) and were sorted by flow cytometry for further experiments. After CD3+/CD8+ and CD4+/CD25+ T cells were treated with EVs/NC or EVs/IL6, cells were labeled with Annexin-V/FITC and PI (Solarbio Life Sciences) for 30 min at the controlled temperature of 37°C and atmosphere of 5% CO_2_; then, the apoptotic rate of cells were detected by flow cytometry.

### 2.15. Statistical Analysis

Statistical analysis was performed using GraphPad Prism 6 V6.0 software (La Jolla, CA, USA). Statistical differences between the two groups were analyzed using Student's *t*-test. The comparison between multiple groups was made using one-way ANOVA test followed by post hoc test (least significant difference). Independent experiments were repeated at least three times for each experiment, and error bars were mean ± standard deviation (*x̅*±SD). *p* < 0.05 was considered statistically significant.

## 3. Results

### 3.1. IL6 Mediates Excessive ROS Production in EC Cells

**Figure 1:**
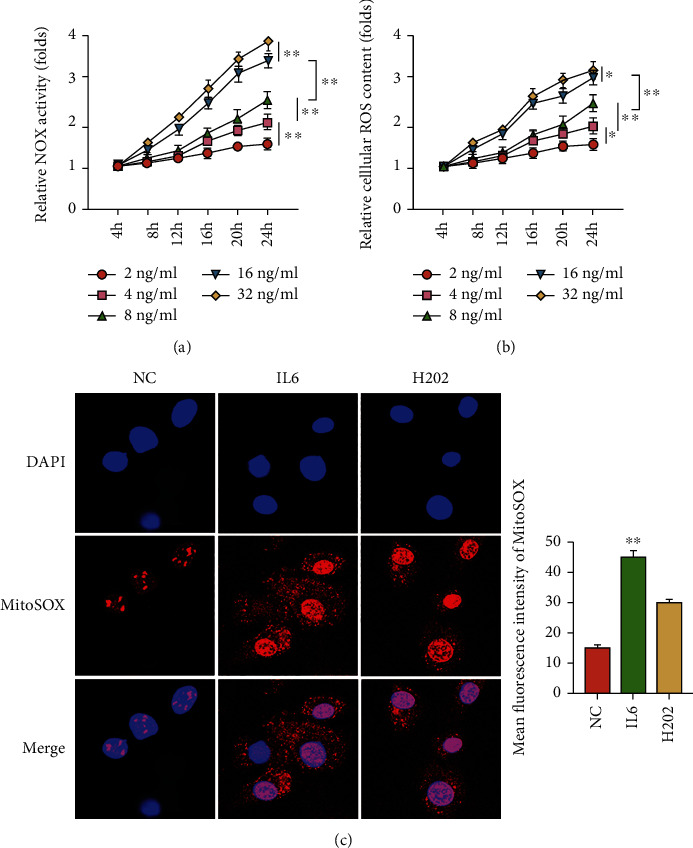
IL6 mediated excessive ROS production in EC cells. (a) The NOX activity in EC cells in the presence of IL6 treatment (2, 4, 8, 16, and 32 ng/ml) for 4, 8, 12, 16, 20, and 24 hr. The NOX activity was measured and expressed as folds changes to that of untreated control cells (designated as 1). (b) The cellular ROS content in EC cells in the presence of IL6 treatment (2, 4, 8, 16, and 32 ng/ml) for 4, 8, 12, 16, 20, and 24 hr. The cellular ROS was measured and expressed as folds changes to that of untreated control cells (designated as 1). (c) Immunofluorescence staining of mitochondrial superoxide production from IL6-treated EC cells. H_2_O_2_ was considered a positive control. The data were represented as the means ± SD; ^∗^*p* < 0.05 or ^∗∗^*p* < 0.01.

High levels of IL6 were supposed to promote tumor development, and activation of the downstream pathway JAK/STAT3 was commonly associated with increased tumor burden [21, 22]. Here, we first detected the NOX activity which was generally identified as a tumor-promoting factor at different time periods (4 h, 8 h, 12 h, 16 h, 20 h, and 24 h) in IL6-induced EC cells [23]. Interestingly, NOX activity increased with the dose of IL6-treated EC cells (2 ng/ml, 4 ng/ml, 8 ng/ml, 16 ng/ml, and 32 ng/ml), with a significant increase at 16 ng/ml (Figure 1(a)). Further investigation showed that ROS content of IL6-treated EC cells also increased in a dose-dependent manner, with a significant increased at 16 ng/ml (Figure 1(b)). To further determine whether ROS stimulated by IL6 in EC, we performed immunofluorescence staining to detect the ROS production by IL6-induced EC cells. Similarly, the resulted showed that IL6-treated EC cells enhanced the production of ROS (Figure 1(c)). These results indicated that IL6 amplified ROS in EC cells via the contribution of NOX activity enhancement.

### 3.2. IL6 Affects Mitochondrial Damage and mtDNA Leakage

**Figure 2:**
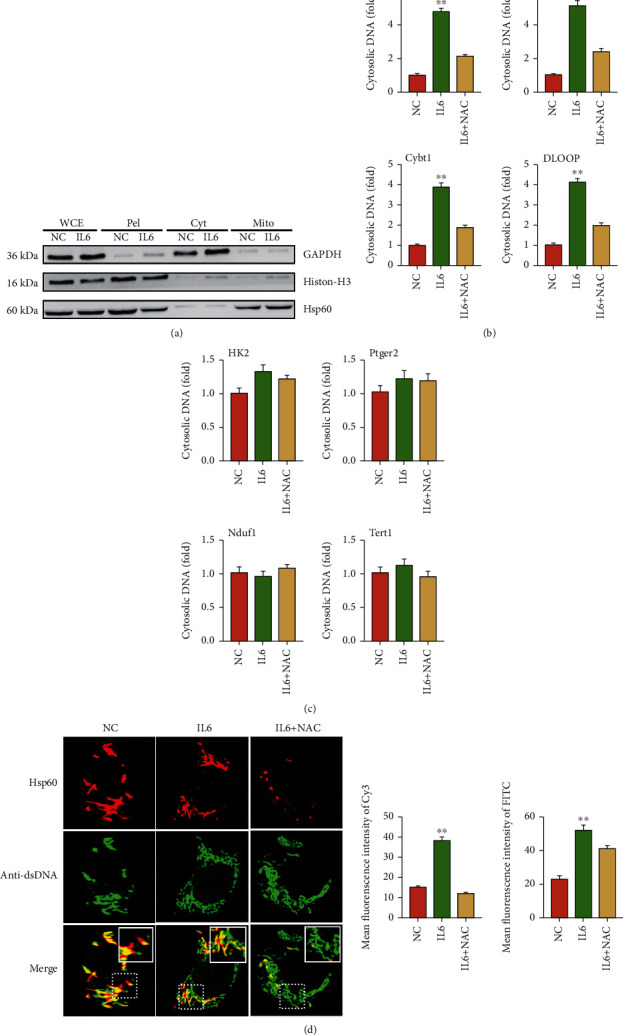
IL6 affects mitochondrial damage and mtDNA leakage. (a) Western blot of whole-cell extracts (WCE), pellets (Pel), cytosolic extracts (Cyt), and mitochondrion (Mito). (b) qRT-PCR analysis of cytosolic DNA (mtDNA) treated with NC, IL6, or IL6 and NAC, respectively. (c) qRT-PCR analysis of cytosolic DNA (nuclear DNA) treated with NC, IL6, or IL6 and NAC, respectively. (d) Immunofluorescence staining of mtDNA (red), and dsDNA (green) treated with NC, IL6, or IL6 and NAC, respectively. The data were represented as the means ± SD; ^∗^*p* < 0.05 or ^∗∗^*p* < 0.01.

To explore the influence of IL6-induced excessive ROS on tumor growth, we isolated EC cells and EC cells treated with IL6 and then purified DNA from cytoplasmic extracts. The fractions were confirmed by western blotting analysis, in which GAPDH, Histone-H3, and Hsp60 represented cytoplasmic, nuclear, and mitochondrial markers, individually (Figure 2(a)). qPCR analysis showed that IL6 treatment increased the mtDNA expression (ND1, ND4, Cybt1, and DLOOP) of EC cells (Figure 2(b)), while no significant differences were observed in the expression of nuclear DNA (Hk2, Ptger2, Nduf1, and Tert1) (Figure 2(c)), suggesting an elevation of cytosolic mtDNA after IL6 treatment. However, these effects were markedly blocked after the coincubation of N-acetyl cysteine (NAC), an inhibitor of ROS generation [24]. Similarly, immunofluorescence staining showed that IL6 increased the content of cytosolic dsDNA which were mainly composed of mtDNA, while treatment with NAC abrogated this effect (Figure 2(d)). These results suggested that IL6 contributed to mtDNA leakage via increasing ROS generation.

### 3.3. IL6 Activates IRF3 through the cGAS-STING Pathway

**Figure 3:**
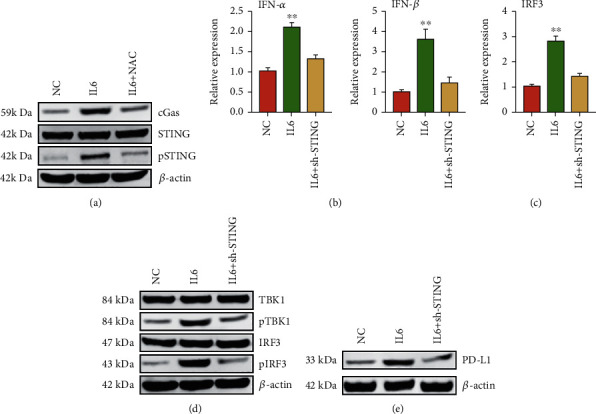
IL6 activates IRF3 through the cGAS-STING pathway. (a) Western blot of cGAS, STING, and pSTING treated with NC, IL6, or IL6 and NAC, respectively. (b) qRT-PCR analysis of IFN-*α* and IFN-*β* treated with NC, IL6, or IL6 and sh-STING, respectively. (c) qRT-PCR analysis of IRF3 treated with NC, IL6, or IL6 and sh-STING, respectively. (d) Western blot of TBK1, pTBK1, IRF3, and pIRF3 treated with NC, IL6, or IL6 and sh-STING, respectively. (e) Western blot of PD-L1 treated with NC, IL6, or IL6 and sh-STING, respectively. The data were represented as the means ± SD; ^∗^*p* < 0.05 or ^∗∗^*p* < 0.01.

To investigate the impact of mtDNA leakage in EC cells, we performed western blot to determine the activation of cGAS-STING pathway, which was activated by excessive DNA content in cytoplasm [15]. The results showed that IL6 increased the protein expression of cGAS and the phosphorylation of STING, while treatment with NAC abrogated this effect (Figure 3(a)). Interestingly, qPCR analysis showed that the expressions of IFN-*α* and IFN-*β* were increased upon IL6 treatments (Figure 3(b)). Besides, the upstream IRF3 was also significantly upregulated upon IL6 treatment; this increase was blocked after STING knockdown, suggesting that IL6 activated IRF3-IFN signaling through cGAS-STING (Figure 3(c)). Western blot analysis indicated that IL6-induced cells activated TBK1-IRF3 through cGAS-STING (Figure 3(d)). We also found that PD-L1, a downstream gene of IFN, was upregulated in IL6-induced cells using western blot analysis (Figure 3(e)). This evidence revealed that IL6-induced mtDNA leakage activated TBK1-IRF3-IFN signaling and increased PD-L1 expression.

### 3.4. IL6-Treated EC Cells Inhibited the Immune Function of T Cells through an EV Transmission Manner

**Figure 4:**
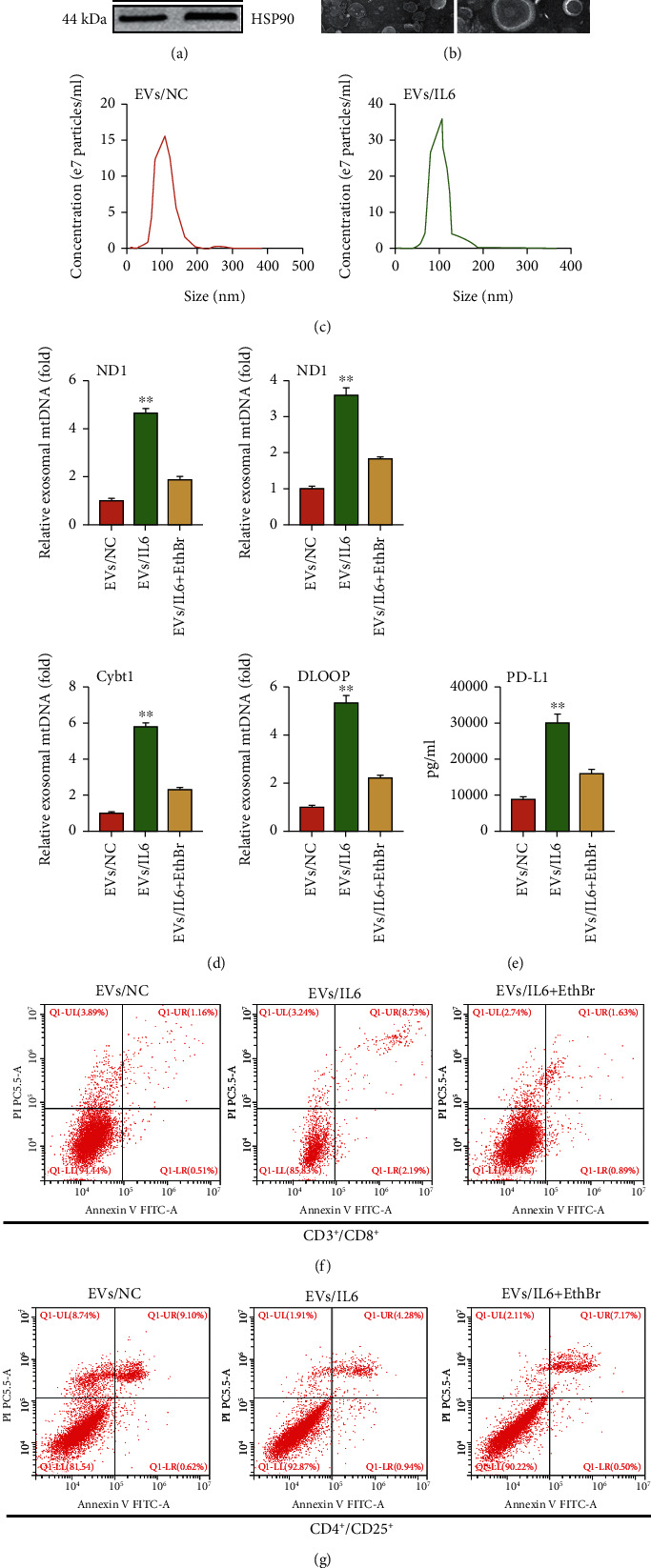
IL6-treated EC cells inhibited the immune function of T cells through an EV transmission manner. (a) Western blot analysis of EV markers (CD9, TSG101, Alix, and Hsp90) in controls or IL6 treatment cells lysate and separated EVs, respectively. (b) Transmission electron microscopy of EVs derived from controls or IL6 treatment, respectively. (c) Nanoparticle tracking analysis of EVs derived from controls or IL6 treatment, respectively. (d) qRT-PCR analysis of cytosolic DNA (mtDNA) in EVs derived from controls, IL6 treatment, or IL6 and EthBr treatment, respectively. (e) The PD-L1 content in purified EV derived from controls, IL6 treatment, or IL6 and EthBr treatment was determined by ELISA, respectively. (f) T cells were treated with the purified EVs derived from controls, IL6 treatment, or IL6 and EthBr treatment, respectively. CD3+/CD8+ cells were measured by flow cytometry. (g) T cells were treated with the purified EVs derived from controls, IL6 treatment, or IL6 and EthBr treatment, respectively. CD4+/CD25+ cells were measured by flow cytometry. The data were represented as the means ± SD; ^∗^*p* < 0.05 or ^∗∗^*p* < 0.01.

Studies have shown that PD-L1 disrupt T cell function through a manner of EV horizontal transfer [19]. Here, we also detect the enrichment of PD-L1 in EC-derived EVs. We first examined the EV production upon IL6 treatment; the results showed that the expression of several EV markers (CD9, TSG101, Alix, and HSP90) were increased upon IL6 treatment (Figure 4(a)). In consistency, TEM analysis and nanoparticle tracking analysis demonstrated an increased secretion of EVs after IL6 treatment (Figures 4(b) and 4(c)). Next, we performed qPCR analysis to determine whether mtDNA was enriched in these vesicles; the result showed that compared with controls, the mtDNA content (ND1, ND4, Cybt1, and DLOOP) was significantly increased in IL6-induced EVs (EVs/IL6) (Figure 4(d)). Importantly, ELISA detection of EVs showed that the level of PD-L1 markedly increased after IL6 treatment (Figure 4(e)). To explore the correlation between mtDNA and PD-L1 carried by EVs and T cell function, we depleted cellular mtDNA by culturing cells in 100 ng/ml ethidium bromide (EthBr). We treated CD4+/CD25+ and CD3+/CD8+ T cells with EVs/IL6 or EVs/IL6 plus EthBr and performed flow cytometry analysis to investigate the cell apoptosis after treatment. CD3+/CD8+ T cell is a main cell population of cytotoxic T cell capable of killing various types of tumor cells, while CD4+/CD25+ T cell is considered a kind of regulatory T cell (Treg) inhibiting the activation of cytotoxic T cell [25]. The results showed that EVs/IL6 promoted the apoptosis of CD3+/CD8+ T cells, while this effect was attenuated by EthBr treatment (Figure 4(f)). In contrast, the apoptotic rate of CD4^+^/CD25^+^ T cells was decreased upon IL6 treatment, which also can be blocked by EthBr treatment (Figure 4(g)). The above results implied that IL6-treated EC cells inhibited the immune function of T cells through a manner of EV transmission.

### 3.5. Masking of PD-L1 in EVs Increased CD3+/CD8+ T Cell Function in Tumor Environment

**Figure 5:**
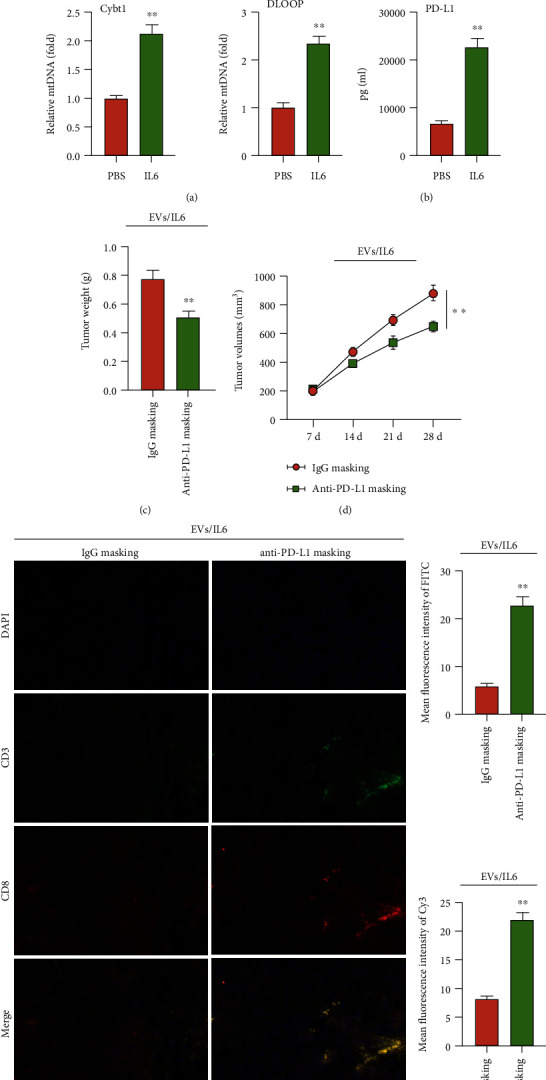
IL6 contribute to immune escape via promote PD-L1 on EC cells-derived EVs *in vivo*. (a) qRT-PCR analysis of cytosolic DNA (mtDNA) in mouse serum EVs treated with PBS or IL6 treatment, respectively. (b) The PD-L1 content in mouse serum EVs treated with PBS or IL6 treatment was determined by ELISA, respectively. (c) Tumor volume was calculated every 7 days after injection treated with EVs/IL6 or anti-PD-L1-masking EVs/IL6. (d) Tumor weight was calculated after tumor excision EVs/IL6 or anti-PD-L1-masking EVs/IL6. (e) Immunofluorescence staining of CD3 and CD8 in resected tumor of mice EVs/IL6 or anti-PD-L1-masking EVs/IL6. The data were represented as the means ± SD; ^∗^*p* < 0.05 or ^∗∗^*p* < 0.01.

We next investigated the effect of IL6 on the progression of EC *in vivo*. To achieve this, we established a mouse xenograft model by subcutaneously injecting MFE-296 cells to C57BL/6 mice. After two weeks, mice were intravenously treated with either PBS or IL6 every 7 days for 28 days. Then, mouse serum was extracted for further assessment. The results showed that compared with the controls, the mtDNA components were significantly upregulated in mouse serum upon IL6 treatment (Figure 5(a)). We also extracted the circulation EVs and found that vesicular PD-L1 level was also increased after IL6 treatment (Figure 5(b)). To further determine the contribution of vesicular PD-L1 in immune escape, we used PD-L1 antibodies (anti-PD-L1) to mask PD-L1 on the surface of EVs/IL6, and xenograft mice were subcutaneously injected with EVs/IL6 or anti-PD-L1-masking EVs/IL6 every 7 days for 28 days. The results showed that either tumor volume or tumor weight was significantly decreased in mice treated with anti-PD-L1-masking EVs/IL6 (Figures 5(c) and 5(d)). Importantly, the mean fluorescence intensity of either CD3 or CD8 were significantly reduced after EVs/IL6 were masked with anti-PD-L1, suggesting that the reduction of vesicular PD-L1 level activated the CD3+/CD8+ T cell function in tumor environment (Figure 5(e)). Together, these findings demonstrated that IL6 contributed to immune escape by increasing the PD-L1 expression of EC-derived EVs, while masking of vesicular PD-L1 rescued the loss of CD3+/CD8+ T cell function.

## 4. Discussion

EC was a common gynecological cancer with various causes for its deterioration. Inflammation was a key driver of tumorigenesis, which may contribute to EC progression. Studies have shown that TNF-*α* was associated with a poor prognosis of EC [26]. Besides, the IL6/STAT3 signaling pathway has been revealed to promote the migration and invasion of EC by upregulating MMP2 expression [27]. In our study, we found that IL6 contributed to immune escape, which ultimately led to EC deterioration.

As an early discovered cytokine, IL6 plays a vital role in chronic inflammation, autoimmune diseases, cancers, and cytokine storms [28, 29]. In cancers such as prostate cancer and liver cancer, the activation of the classic IL6 signaling pathway is generally accompanied by tumor progression, which can be reversed by inhibition of the IL6/JAK/STATS pathway [30, 31]. Moreover, IL6 also can regulate tumor progression through other pathways. Previous studies have demonstrated that in human abdominal aortic aneurysms, IL6 increased NOX activity and MALAT1 expression in a time-dependent manner, leading to excessive ROS production. The excess ROS, in turn, increases the tumor burden [32, 33]. We found a similar phenomenon in EC that IL6 induced excessive ROS production. In our study, we focused on ROS-mediated mitochondrial damage and discovered that the mtDNA produced by ROS damage was secreted by EVs. This phenomenon was consistent with a previous study [34].

mtDNA is a unique type of mitochondrial DNA that typically produces a stress response to radiation, toxic chemicals, infection by pathogenic microorganisms, or an excess of ROS. Mitochondria release content that contains mtDNA to cope with stress damage [35]. In our study, we also found that ROS produced by IL6-treated EC cells resulted in the leakage of large amounts of mtDNA from mitochondria into the cytoplasm. In EC, the leaked mtDNA significantly increased the content of dsDNA and then activated the downstream cGAS-STING pathway.

The cGAS-STING pathway has been found to be an important DNA sensing mechanism in innate immunity and virus defense. Its activation usually activated the production of type I IFN and triggers innate immunity against cancer. Therefore, cGAS-STING agonists have been used in the treatment of cancers [36]. STING agonists stimulated the pancreatic cancer immune microenvironment and resist tumor progression in mouse models [37]. However, increasing evidence suggested that it also accelerates tumor growth and metastasis on the context dependent. Its chronic activation paradoxically induces an immunosuppressive tumor microenvironment [15]. A previous study has shown that overexpressed Lon cells secreted EVs with mtDNA and PD-L1. These EVs attenuated T cell immunity in TME by inducing macrophages to produce IFN and IL6 [38]. In our study, we also found the ROS-mediated leakage of mtDNA increases the secretion of EVs in EC cells. These EVs, which contain PD-L1 and mtDNA, weaken antitumor immunity and contribute to tumor survival.

Our study had several limitations. Firstly, we just used an EC cell (MFE-296) to finish the in vitro and in vivo experiments. More EC cells are needed to further confirm our findings. Secondly, the specific mechanisms involved in IL6 inducing mtDNA leakage have not been investigated.

In conclusion, our study found that IL6 induced large amounts of ROS through NOX in EC. ROS damage mitochondrial function and lead to mtDNA leakage, thereby elevating dsDNA. The leaked mtDNA increased the expression of type I INF and PD-L1 through the cGAS-STing-TBK1-IRF3 axis. Finally, PD-L1 and mtDNA were assembled into EVs and interact with T cells leading to tumor immune escape. Hence, our study reveals a novel mechanism by which IL6 increases EC burden and mediates EC cell immune escape.

## Data Availability

The datasets used and/or analyzed during the current study are available from the corresponding authors on reasonable request.
